# Autohomogenization of Polybenzimidazole Composites with Enhanced Mechanical Performance by Air Incorporation

**DOI:** 10.1021/acs.langmuir.4c02745

**Published:** 2024-10-31

**Authors:** Jiabei Zhou, Xianzhu Zhong, Kenji Takada, Maiko K. Okajima, Masayuki Yamaguchi, Tatsuo Kaneko

**Affiliations:** 1 Graduate School of Advanced Science and Technology, Japan Advanced Institute of Science and Technology (JAIST), 1-1 Asahidai, Nomi 923-1292, Japan; 2 Key Laboratory of Synthetic and Biological Colloids, School of Chemical and Material Engineering, Jiangnan University, 1800 Lihu Ave., Wuxi 214122, China; 3 Graduate School of Organic Materials Science, Yamagata University, 4-3-16, Jonan, Yonezawa 992-8510, Japan

## Abstract

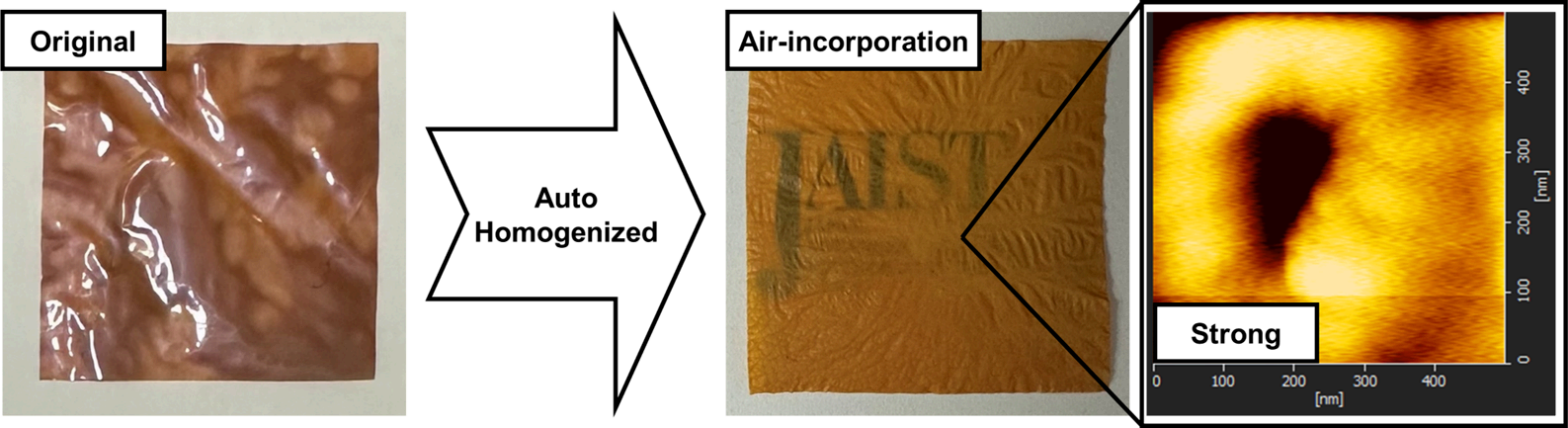

Polybenzimidazoles are one of the most thermally and chemically stable polymers due to their rigid chemical structure with π–π stacking and conjugated bonding. Poly(2,5-benzimidazole) (ABPBI), the simplest structure of polybenzimidazole, was synthesized, but the cast film was not homogeneous and featured thick brown areas, which limited their further application. Silica nanospheres were adapted as porogen to generate nanopores in the ABPBI film by successive etching with hydrofluoric acid. As a result of air-composite formation, the ABPBI film became homogeneous and its surface roughness was reduced from 10.0 to 2.5 nm. The obtained air-composite ABPBI film had more favorable mechanical properties than the original film. An air-composite film prepared with 50 wt % silica content had a tensile strength of 128 MPa and an elongation at break of 23%, both of which values were approximately twice as high as the corresponding values of the original film.

## 1 Introduction

Porous materials play a crucial role as air composites in modern industry owing to their unique properties. These include low bulk densities, high surface areas, low dielectric constants, the ability to act as molecular nanocontainers, and damping capabilities. Moreover, they are useful in catalysts, packaging, drug delivery, segregation, and absorption/desorption.^1−5^ Taking inspiration from natural porous materials such as bamboo and honeycomb, various porous materials with diverse structures have been engineered to serve specific purposes.^6−9^ For example, metal–organic frameworks^10−12^ and covalent–organic frameworks^13,14^ with exceptional performances have been designed with intricate porous architectures for the capture and segregation of nitrogen or carbon dioxide, and they have great potential for various applications.^15,16^ However, organic porous materials by their very nature tend to be relatively unstable when exposed to high temperatures. This limitation restricts their use in applications requiring high thermo-resistance, such as the segregation of waste gases in high-power engines.^17^ Furthermore, many existing porous materials, including metal– and covalent–organic frameworks, are derived from fossil-fuel-based resources, raising concerns about their sustainability as petroleum resources become depleted. Therefore, there is a pressing need to develop porous materials with high thermo-resistance using natural renewable resources. This endeavor is important for ensuring the long-term sustainability of porous materials in various industrial applications.^18−23^

Polybenzimidazole (PBI) is known for its exceptional thermal stability, chemical durability, and outstanding mechanical properties. It is therefore an excellent candidate for applications that require heat-resistant materials.^24−29^ Conventional PBI is used widely in numerous applications owing to its high thermo-resistance. However, its precursor monomer is available only from petroleum-based resources. This poses a significant challenge to the development of conventional PBI from biobased sources. After a series of chemical modifications to address this challenge, 3,4-diaminobenzoic acid (34DABA) was obtained from bioresources.^30^ This breakthrough made producing a biobased conventional PBI variant, known as poly(2,5-benzimidazole) (ABPBI), feasible. Owing to its unique structural distribution of highly aromatic conjugation, ABPBI has remarkable thermal stability, with a 10% degradation temperature surpassing 600 °C. This places it ahead of conventional PBI in terms of heat resistance. However, casting ABPBI films remains a challenge, owing to their nonhomogenous texture caused by ABPBI’s tendency to aggregate into irregularly branched islands. This results in unstable mechanical properties and low durability and limits the usefulness of the material.

Herein, we created a porous film by mixing ABPBI with a silica porogen and subsequently etching the silica. The process had two results: (1) the surface roughness of the film was reduced by incorporating the silica and (2) the pore structures were effective in increasing the mechanical toughness of the film compared with the original pure film. Given its high thermal stability and bioavailability, we anticipate that the porous ABPBI film will be useful in industrial applications.

## 2 Experimental Section

### 2.1 Materials

3,4-Diaminobenzoic acid (34DABA, 99% purity) and phosphorus pentoxide/methanesulfonic acid (P_2_O_5_/CH_3_SO_3_H) (Eaton’s reagent) were purchased from Tokyo Chemical Industry Co., Ltd. Spherical silica particles (seahostar@KE-P30, diameter: 300 nm) were purchased from Nippon Shokubai Co., Ltd., in Osaka, Japan. Hydrofluoric acid (HF, 55% purity) was obtained from Morita Chemical Industries Co., Ltd. Trifluoroacetic acid (TFA), methanesulfonic acid (MSA), sodium bicarbonate, and other chemicals were obtained from Kanto Chemical Co., Inc. All of the chemicals and reagents were used as received.

### 2.2 Measurements

Fourier-transform infrared (FT-IR) spectra were obtained to determine the chemical structure of the synthesized ABPBI. The wavenumbers were in the range of 400–2000 cm^–1^, and the spectra were obtained using a PerkinElmer spectrometer with a diamond-attenuated total reflection (ATR) accessory. Thermogravimetric analysis (TGA) was performed using a HITACHI STA2700 system. The film specimens were placed in a platinum crucible and heated to a maximum temperature of 800 °C at a rate of 10 °C min^–1^ under a nitrogen atmosphere. The morphology of each fabricated film was determined by using a scanning electron microscope (SEM; HITACHI TM3030 plus tabletop SEM). Energy-dispersive X-ray spectroscopy (EDS) was used to identify residual elements in the prepared films. The film specimens were precoated with Au powder under a vacuum for 30 s (thickness: 15 nm). Atomic force microscopy (AFM; HITACHI AFM5000 II SPA-400) was used to determine the morphology and roughness of each film. Tensile tests were carried out using an Instron-3365 mechanical tester instrument at an ambient temperature of around 25 °C. The film samples were cut to an appropriate size (30 mm × 5 mm) and 5 specimens with an initial gauge length of 20 mm were tested at a tensile speed of 5 mm min^–1^.

### 2.3 Synthesis of Polybenzimidazole

3,4-Diaminobenzoic acid (34DABA) (1.521 g, 10 mmol) and 20 mL of phosphorus pentoxide/methanesulfonic acid were added to a three-necked flask equipped with a magnetic stirrer at 25 °C under a nitrogen flow. When 34DABA had dissolved completely, the temperature was increased to 150 °C to initiate polymerization. The viscosity of the solution increased as the polymerization time increased, and the solution changed from yellow to dark brown. After reacting for 24 h, the solution was added to deionized water to precipitate the polymeric fibers. The filtered ABPBI fibers were dried at 100 °C for 24 h in a vacuum and crushed to a powder. After neutralizing with a 5 wt % sodium bicarbonate solution, the ABPBI powder was repeatedly washed with deionized water and dried at 60 °C for 12 h. Here, it is noted that the present ABPBI is derived from commercial 34DABA and then we did not confirm the bioderivation of ABPBI.

### 2.4 Fabrication of Pure ABPBI Film

ABPBI powder (50 mg) was dissolved in a mixture of TFA with two drops of MSA. After stirring at room temperature for 24 h until a homogeneous solution formed, the TFA was evaporated to produce a 1 wt % solution. The ABPBI solution was cast on a silicon wafer substrate to obtain a film. The cast film was dried at room temperature for 12 h and immersed in deionized water to remove the residual acid. After it was washed repeatedly with deionized water, the ABPBI film was dried at 60 °C for 12 h in a vacuum.

### 2.5 Fabrication of Silica Composite ABPBI Film

ABPBI powders (90, 80, 70, 60, and 50 mg) and TFA (3 mL) were added to a screw-capped bottle and magnetically stirred at room temperature for 24 h. After 2 drops of MSA were added and stirred for 24 h at room temperature, the solution became clear. Silica nanospheres (10, 20, 30, 40, and 50 mg) with an average size of 300 nm were ultrasonicated in TFA at room temperature for 1 h to thoroughly disperse them. The ultrasonicated silica dispersion was immediately mixed with the ABPBI solution and further ultrasonicated at room temperature for 1 h just before the film casting. The ultrasonicated mixture was cast onto a silicon wafer to fabricate ABPBI–silica composite films. After the TFA was evaporated at room temperature, the formed ABPBI–silica composite films were washed repeatedly with deionized water and immersed in deionized water for 24 h to completely remove the residual acid. The washed ABPBI–silica composite films were then dried at 60 °C for 12 h in a vacuum.

### 2.6 Fabrication of Porous ABPBI Films

**Figure 1:**
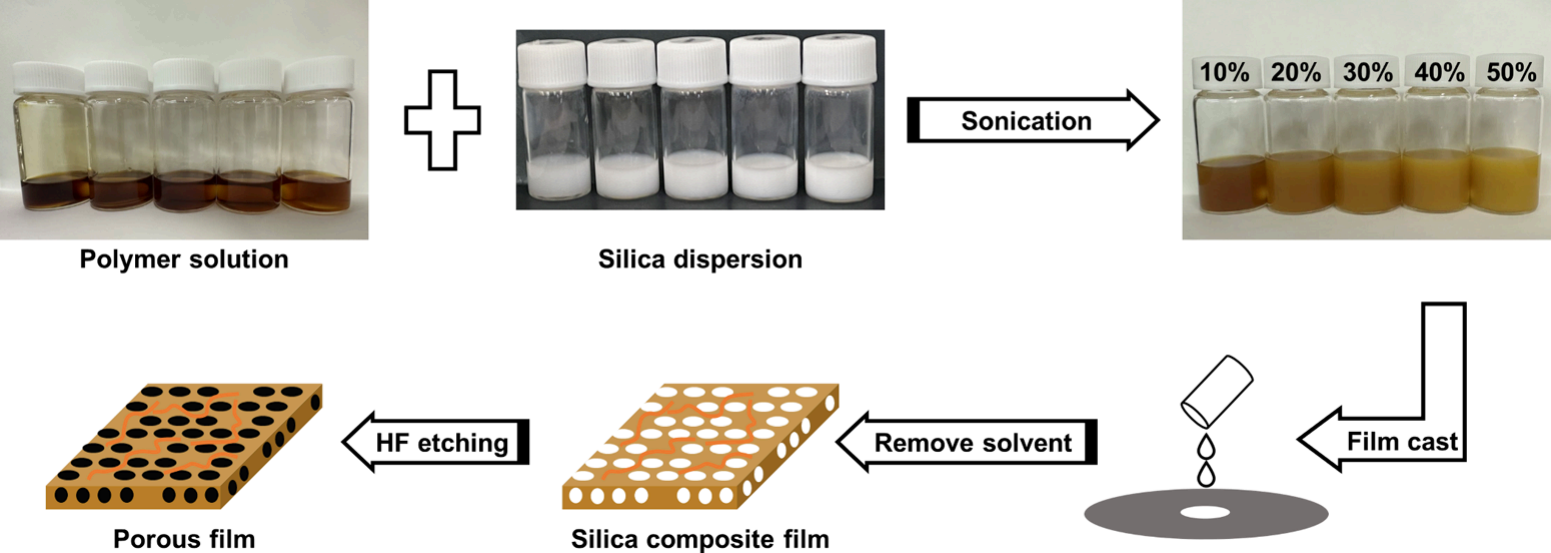
Fabrication of the porous ABPBI films (ABPBI = poly(2,5-benzimidazole)).

The dried ABPBI–silica composite films were immersed in a 40% HF aqueous solution for 24 h to fabricate the porous ABPBI films (Figure 1). After washing repeatedly until the pH reached approximately 7 according to pH test paper, the porous ABPBI films were soaked in deionized water for 24 h to remove residual acid and dried at 60 °C for 12 h in a vacuum.

## 3 Results and Discussion

### 3.1 Preparation of Air Composites

ABPBI is conventionally synthesized by the polycondensation of 34DABA using an acidic solvent of very high viscosity poly(phosphoric acid). The reaction is very time-consuming, and a large amount of water is required to remove the catalyst from the product. Herein, we attempted to improve the efficiency of synthesis by using another reagent, i.e., Eaton’s reagent, which is known as a solvent system for polybenzazole syntheses. The polymerization proceeded smoothly, and solvent removal was completed easily. The chemical structure of the obtained ABPBI was confirmed by FT-IR (ATR mode) and the spectrum is shown in Figure S1. The signals at 1627, 1551, and 1283 cm^–1^ were assigned to the stretching vibrations of the C=N, C=C, and C–N groups, respectively. The FT-IR spectra indicated the successful synthesis of ABPBI using Eaton’s reagent.

**Scheme 1:**
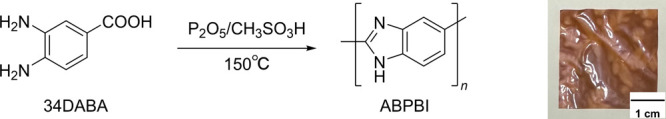
Synthesis of ABPBI from 34DABA Using Eaton’s Reagent Right picture: photo of ABPBI film (ABPBI = poly(2,5-benzimidazole); 34DABA = 3,4-diaminobenzoic acid; Eaton’s reagent = phosphorus pentoxide/methanesulfonic acid (P_2_O_5_/CH_3_SO_3_H)).

The ABPBI powder was processed into a film by a casting method. TFA with a trace amount of MSA was used as the solvent for casting because ABPBI is readily soluble in it. The film was completely dried within 1 h owing to the low boiling temperatures of MSA (122 °C) and TFA (72.4 °C). The resulting film was mechanically robust and brown in color. The film surface was heterogeneous, i.e., featured branched dark brown regions that were a maximum of 30 μm thick separated from the thinner matrix, which was a minimum of 18 μm thick (Scheme 1, right photo). The statistical difference was calculated as 3.06 μm. During drying, the ABPBI solution condensed and was deposited owing to the strong intermolecular interactions of ABPBI, which is highly polar. When the brown regions began to form, the ABPBI around the solution surface became extremely condensed, which induced interchain self-assembly, and the assembled matter absorbed the solvent, thereby becoming sticky. The assembled matter grew into a fibrous aggregate following further drying from the solution surface, whereas the ABPBI around the middle and bottom of the solution was still dissolved, owing to the increased concentration of MSA in the mixed solvent. Ultimately, thick brown fibrous branches remained in the cast film. Unfortunately, such heterogeneity limits the usefulness of the ABPBI film.

**Figure 2:**
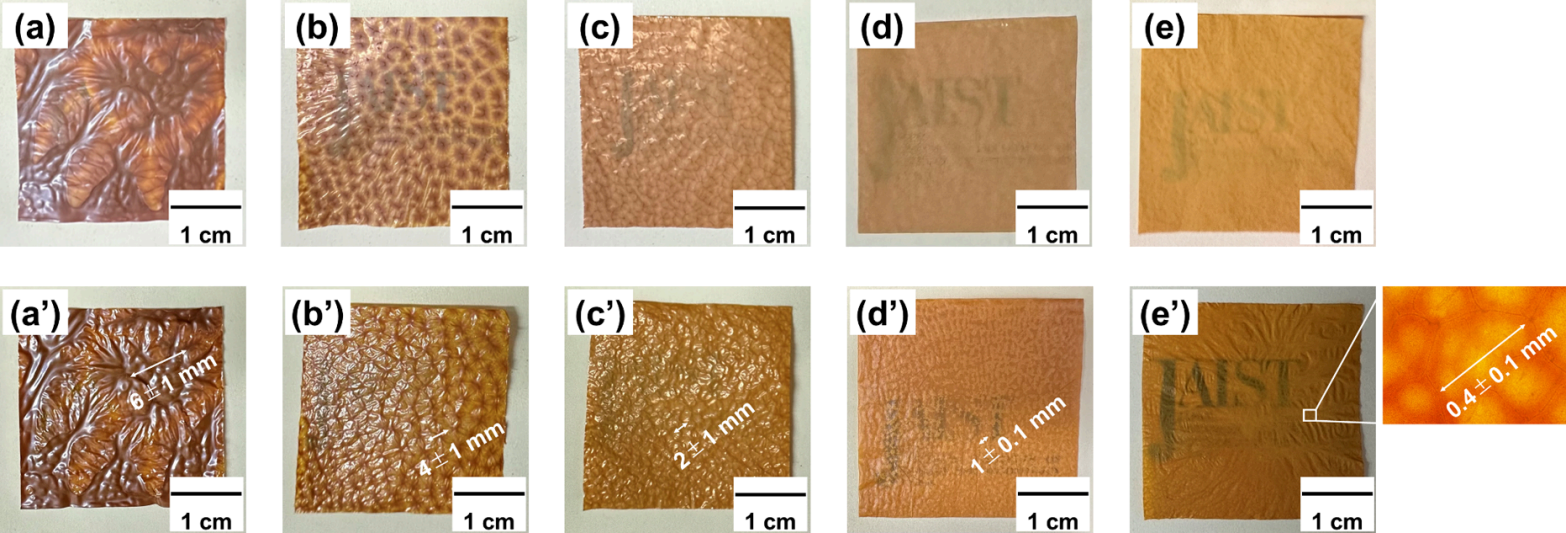
Photos of ABPBI composite films with silica components of (a) 10, (b) 20, (c) 30, (d) 40, and (e) 50% and those with air components of (a′) 8, (b′) 16, (c′) 24, (d′) 32, and (e′) 40%. The air components were calculated from the silica density. The inset photo of (e′) is an optical microscope image (ABPBI = poly(2,5-benzimidazole)).

We prepared air composites of ABPBI by a silica-etching method. We used ultrasonication to disperse the silica nanospheres as uniformly as possible in the ABPBI before casting the films. Consequently, the silica dispersion coexisted stably with ABPBI for more than 1 h. The aim was to form a homogeneous ABPBI film containing silica nanospheres. However, the resulting films were firm, but heterogeneity remained. As shown in Figure 2a′, the thick brown regions were wrinkled and more branched than in the original ABPBI film without silica. The distance between the wrinkles was 6 ± 1 mm, and the size and distance between them decreased as the amount of silica increased, as shown in Figure 2a′–e′. The thick brown regions decreased in size from 6 to 0.4 mm when the amount of silica nanospheres increased from 10 to 50 mg. The branched wrinkles almost disappeared when the weight of the silica nanospheres matched that of the ABPBI. We mixed the silica nanospheres with the ABPBI to act as porogens; the results revealed that they acted as homogenizers to produce uniform films of ABPBI. The dispersion mechanism is thought to involve a strong interaction between the surfaces of the silica nanosphere and the ABPBI chains caused by their high polarity and favorable dispersion behavior. When the silica nanospheres were stably dispersed in the films by ultrasonication, the ABPBI chains adhered to the particle surfaces were distributed uniformly.

We used an aqueous solution of HF to remove the silica nanospheres from the silica–ABPBI films to fabricate the air–ABPBI composite films. The air content was calculated from the added silica nanosphere content using the density of silica, as shown in Figure S2. FT-IR/ATR spectroscopy revealed no peak-shifting following the silica-etching treatment of the original ABPBI films to produce the porous films, demonstrating that HF etching did not cause any chemical damage, owing to the high chemical stability of ABPBI. Figure 2 demonstrates that the patterns of the thick brown regions were almost retained after the silica-etching treatment, although the film transparency increased, suggesting that the treatment appropriately dissolved out the silica particles without causing any other film damage.

### 3.2 SEM Morphologies

**Figure 3:**
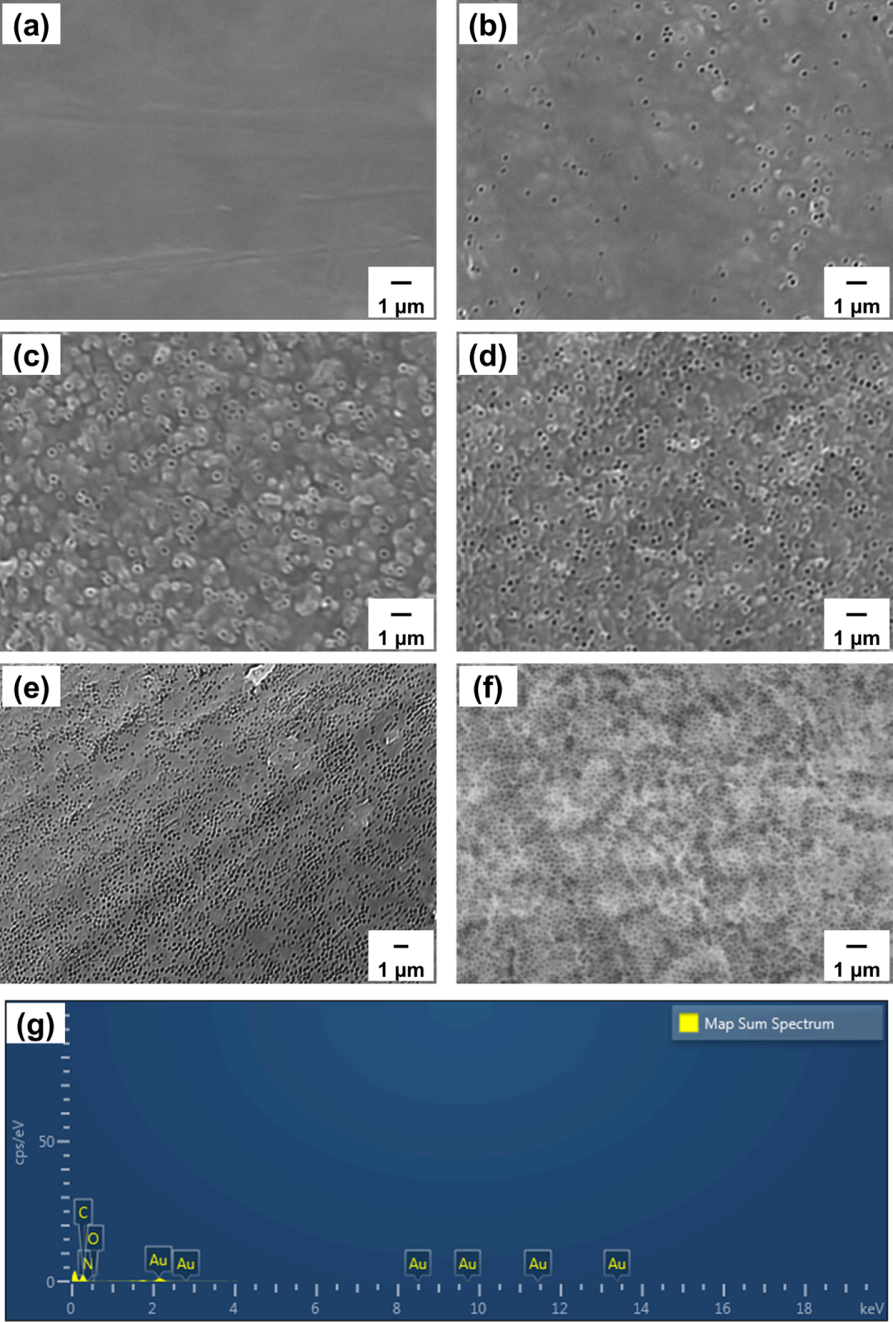
SEM images of original (a) and porous ABPBI films with air components of (b) 8, (c) 16, (d) 24, (e) 32, and (f) 40%. (g) EDS elemental analysis of (f) (SEM = scanning electron microscopy; ABPBI = poly(2,5-benzimidazole); EDS = energy-dispersive X-ray spectroscopy).

We used SEM to investigate the morphologies of the obtained air composites (Figure 3). The pure ABPBI film had a flat surface (Figure 3a), whereas all of the air-incorporated films had pores on their surfaces. It was revealed from the SEM images that the increased porosity caused by increasing the amount of silica in the composition (Figure 3b–f). Moreover, it indicates the successful preparation of ABPBI films with tailored porosities. Additionally, we calculated the volume percentage of the film porosity by the 3/2 power of area percentage estimated from SEM images to be 9, 16, 26, 34, and 43%, showing a good coincidence with the calculated results of air components from the added silica content. The EDS map reveals that no elemental silicon or fluorine remained, suggesting that the silica nanospheres were completely removed by HF and the HF solution was completely washed out (Figure 3g and Figures S4–S7).

**Figure 4:**
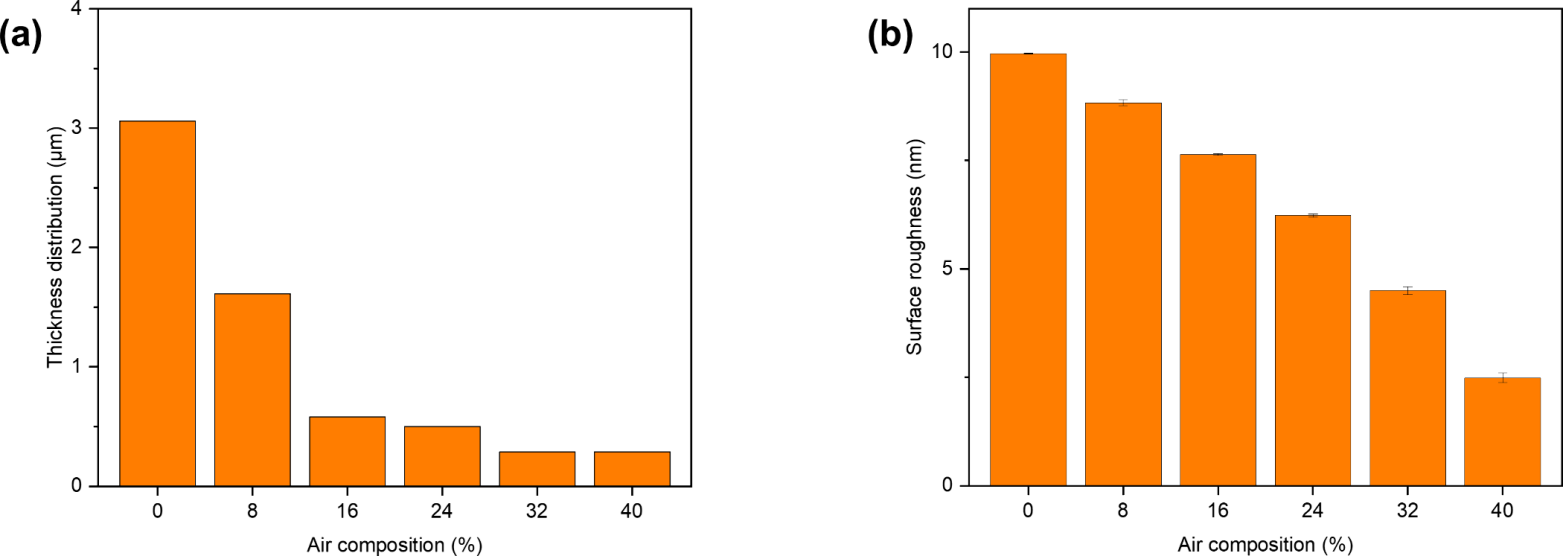
Thickness distribution (a) and surface roughness on the nanoscale (b) of ABPBI films with various air compositions (ABPBI = poly(2,5-benzimidazole)).

The thickness difference of the dark brown region on the obtained air-composite ABPBI films was measured using a micrometer gauge and summarized as a thickness distribution (Figure 4a). The thickness distribution which was calculated from the thickness variations, was quantified as 3.1 to 0.3 μm and decreased as the amount of silica increased from 10 to 50%, confirming that silica-etching treatment reduced the surface roughness of the material. AFM analyses also revealed a decrease in the roughness of the air-composite surface on the nanoscale (i.e., 10–2.5 nm) (Figure 4b). The findings indicate that the assembled materials could be readily controlled by adding homogeneously dispersed silica nanospheres.

### 3.3 AFM Analysis

**Figure 5:**
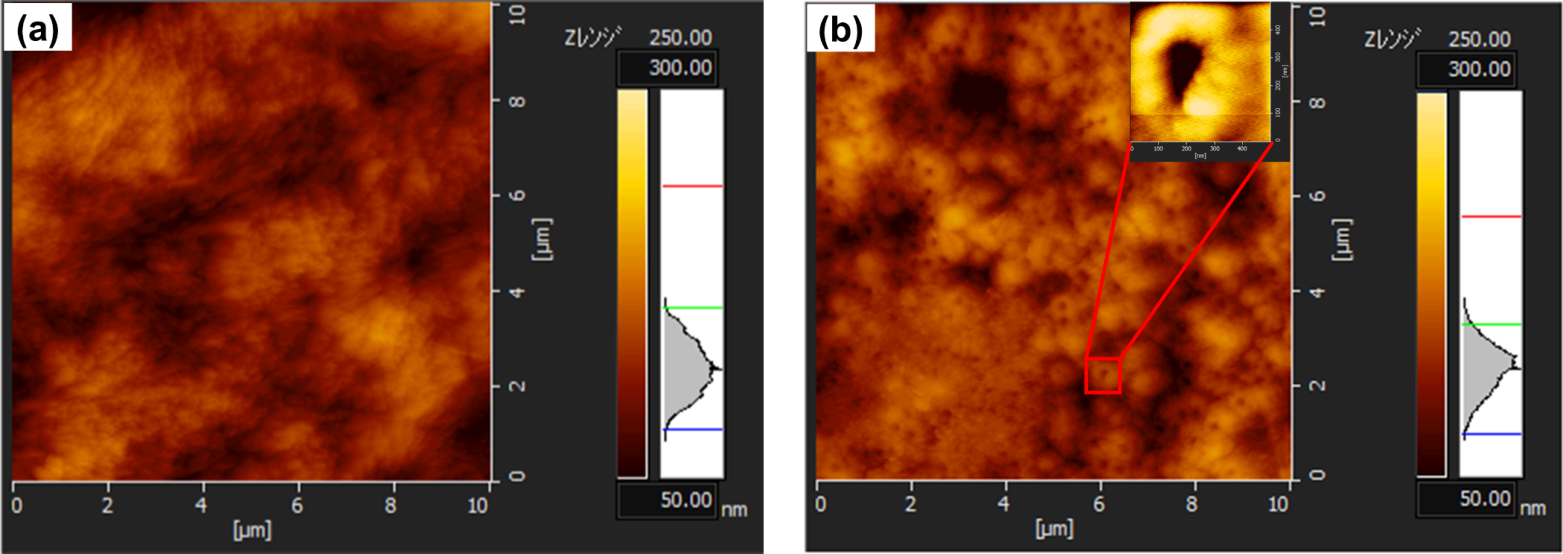
AFM images of ABPBI films with air compositions of (a) 0 and (b) 16% (inset photo: single pore) (AFM = atomic force microscopy; ABPBI = poly(2,5-benzimidazole).

The SEM images revealed pores with white edges, which inspired us to envision the formation of a crater-like morphology. Hence, we subsequently investigated the pore morphology by AFM, using ABPBI films with air compositions of 0 and 16% (Figure 5). The original ABPBI film had a rough surface and produced a broad height distribution curve (Figure 5a), whereas the ABPBI film with an air composition of 16% had a more homogeneous surface and produced a much narrower height distribution curve (Figure 5b). It indicated that the roughness was reduced by the silica-etching treatment. The inset of Figure 5b is the enlarged image of a single pore with a diameter of approximately 300 nm, corresponding with the diameter of the silica nanospheres. Linear height analysis was used to investigate the height difference in the circle (Figure S3). The highest areas, which were approximately 50 nm high, were located around the pores. Therefore, the circular edges of the pores were swollen like craters. The number of craters increased as the silica nanosphere content increased. Crater formation was also confirmed in films comprising an ABPBI copolymer with 4-aminobenzoic acid.^31^ The ABPBI chains aggregated on the silica nanosphere surfaces, as discussed above, and the aggregated chains remained during the solvent evaporation process.

### 3.4 TGA Analysis

**Figure 6:**
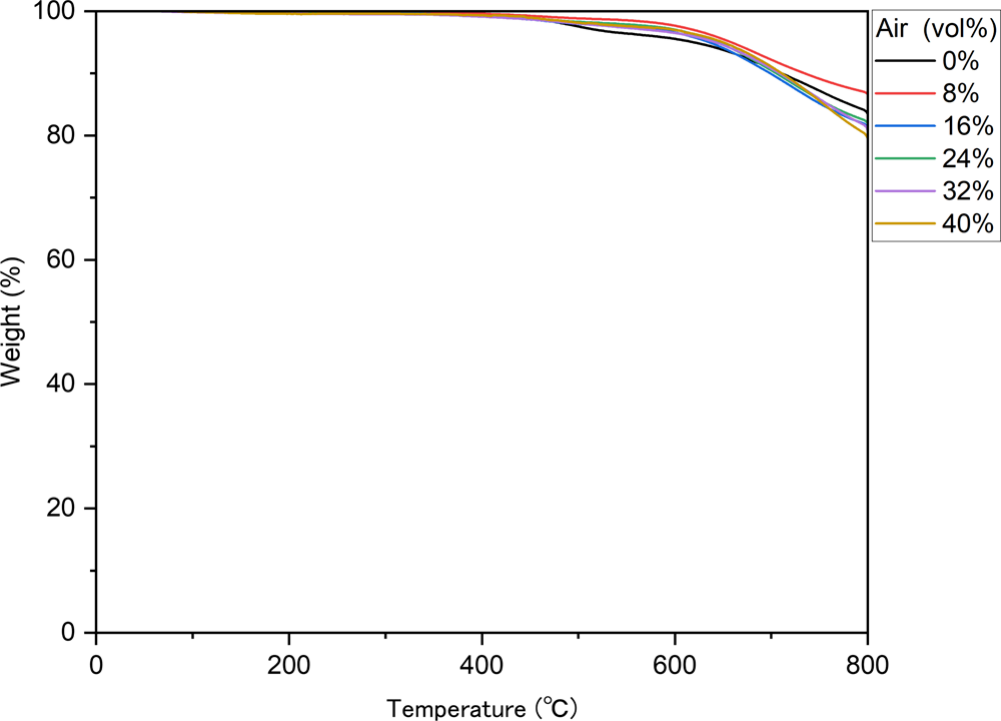
TGA curves of ABPBI films with various air contents (TGA = thermogravimetric analysis; ABPBI = poly(2,5-benzimidazole)).

Thermogravimetric analysis (TGA) was used to assess the thermal characteristics of ABPBI films with various air contents (Figure 6). The TGA results revealed a constant value of approximately 100% mass from 0 to 400 °C, demonstrating the high thermal stability of the original ABPBI film and complete ring-closure to form an imidazole ring during the synthesis process. Furthermore, the resultant ABPBI had an exceptionally high 10% mass-loss temperature (*T*_d10_: 710 °C), which was comparable to those of other ABPBI polymers synthesized using the conventional poly(phosphoric acid) method. This suggests that Eaton’s reagent is useful for synthesizing ABPBI. Moreover, regardless of their expanded surface areas, the porous ABPBI films had high *T*_d10_ values of over 700 °C and high char yields of approximately 80%, as shown in Table 1. They are therefore potentially useful for many applications, such as ultrahigh thermoresistant porous plastics.

**Table 1:** Thermal and Mechanical Properties of the Porous ABPBI Films with Various Air Contents^c^

air composition	*T*_d10_^a^ (°C)	char yield^a^ (%)	Young’s modulus^b^ (GPa)	strength at break^b^ (MPa)	elongation at break^b^ (%)	strain energy density^b^ (MJ/m^3^)
ABPBI-0	710	84	1.34 ± 0.05	59 ± 5	9 ± 1	3.9 ± 0.3
ABPBI-8	735	87	1.09 ± 0.05	36 ± 5	3 ± 1	0.5 ± 0.2
ABPBI-16	700	81	0.71 ± 0.04	27 ± 4	7 ± 1	1.0 ± 0.2
ABPBI-24	705	82	1.30 ± 0.03	45 ± 3	11 ± 1	3.6 ± 0.1
ABPBI-32	710	81	1.92 ± 0.03	62 ± 2	12 ± 1	5.8 ± 0.1
ABPBI-40	710	80	2.48 ± 0.02	128 ± 2	23 ± 2	21.6 ± 0.1

a Thermal property obtained by thermogravimetric analysis (TGA).

b Mechanical properties of the porous ABPBI films obtained from uniaxial tensile tests. The value of standard deviations was calculated from the mechanical test data of five specimens.

c ABPBI = poly(2,5-benzimidazole).

### 3.5 Stress–Strain (S–S) Curves

**Figure 7:**
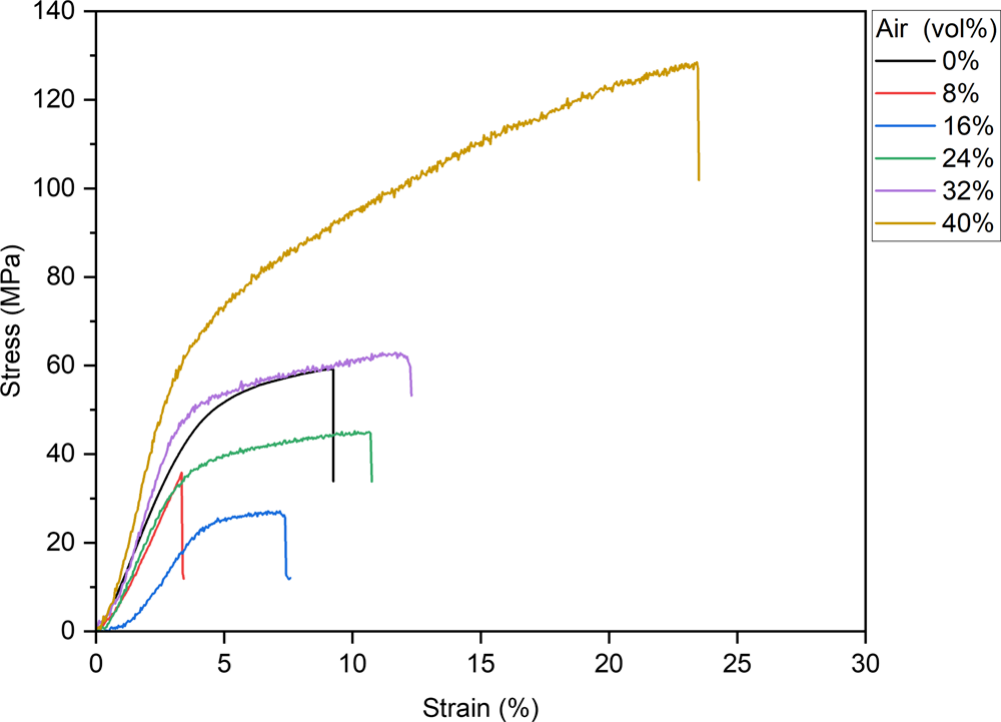
S–S curves of ABPBI films with various air contents (S–S = stress–strain; ABPBI = poly(2,5-benzimidazole)).

The mechanical properties of the ABPBI composite films were assessed by examining their stress–strain curves. The silica composite films were too brittle for a valid investigation, so we focused on the air-composite films (Figure 7). When a small number of pores were incorporated, the mechanical properties such as strength at break, elongation at break, Young’s modulus, and strain energy density decreased. However, the mechanical properties were gradually improved as the air content increased. When the air content reached 32%, the stress–strain curve became comparable to that of the original ABPBI film. Finally, when the air content was 40%, the highest value of the strength at break was 128 MPa, and the elongation at break was 23%, both of which values exceeded those of the original nonporous ABPBI film: 59 MPa for the strength at break and 9% for the elongation at break, demonstrating a significant improvement in the mechanical performance. Moreover, the strain energy density increased to 21.6 MJ m^–3^, i.e., almost 5 times as high as that of the original ABPBI film, with a value of 3.9 MJ m^–3^. Despite the presence of air, Young’s moduli of the films with air contents of 32 and 40% exceeded that of the original ABPBI film. The enhanced mechanical properties could be attributed to the synergistic effect between the mechanical decrease effect from the air area and the mechanical increase effect from the crater-like area, which resulted from the surface energy of silica nanospheres. As for the air-composite films with low air content, the mechanical decrease effect from the air area is stronger, showing a more brittle behavior than the original ABPBI film, although a small number of hard craters were fabricated. Subsequently, as the air content improved, the mechanical increase effect was enhanced along with the number of hard craters raised, indicating an increased mechanical property. On the other hand, at high air content, the pores with hard craters became connected with each other, constructing a hard architectural network, and the enhanced intermolecular interactions significantly improved the mechanical properties, in comparison with the original ABPBI film. Moreover, the effects of circular aggregates around the crater edges can increase Young’s modulus to improve the film’s strength and toughness. In addition, it can be noted that the standard deviation values shown by plus/minus values of strength at break and strain energy density (toughness) in Table 1 were decreased with an increase in the composition of air and silica nanospheres, demonstrating that the increased homogeneity is effective on the mechanical data accuracy. Therefore, not only the flexibility but also the stiffness was enhanced by air incorporation where the air worked as a softener and the pore with hard craters acted as a hardener, which could be an original conceptualization for the fabrication of the stiff and elastic polymers, consist of rigid backbones and high polarity.

## 4 Conclusions

In this study, we successfully formed an ABPBI film from 34DABA and confirmed its structure by FT-IR analysis. We fabricated ABPBI films by solution casting using TFA with a small amount of MSA as a solvent. The surfaces of the films were heterogeneous and featured branched patterns of thick brown regions, which may have originated from the surface condensation of ABPBI to form sticky fibrous aggregates. Subsequently, we fabricated porous ABPBI films with various air contents by a hard-templating method using silica nanosphere fillers, followed by etching with HF. The successful construction of pores was confirmed by SEM and AFM, which revealed the formation of polymer aggregates, like craters, around the pore edges. The composite ABPBI films had reduced heterogeneity and roughness compared to the original ABPBI film. Herein, we proposed a formation mechanism whereby the ABPBI aggregate stuck to the surfaces of the silica nanospheres, which were well dispersed over the film by ultrasonication. TGA characterization revealed that the ABPBI air composite had a 10% mass loss temperature of 710 °C, indicating that it had an ultrahigh thermal stability that surpassed that of most of the available organic materials. Moreover, the air composition conferred an obvious improvement in mechanical properties owing to the hard aggregates around the pores. This suggests the potential of ABPBI as a porous material for use in, e.g., fuel cell separators, leakproof films, and filtration membranes.

## Supporting Information Available

FT-IR spectra of ABPBI films with various air contents; air composition calculation; linear height analysis; SEM images, EDS mappings, and elemental analysis of porous ABPBI films (PDF)
