# Supplementary material for: Autohomogenization of Polybenzimidazole Composites with Enhanced Mechanical Performance by Air Incorporation
Source: Langmuir. 2024 Oct 31;40(45):23780–7. doi: 10.1021/acs.langmuir.4c02745 (PMC11562793; doi:10.1021/acs.langmuir.4c02745)
Supplement: Supplementary file 1 — la4c02745_si_001.pdf [file la4c02745_si_001.pdf]

Auto-homogenization of Polybenzimidazole Composites with Enhanced Mechanical Performance by Air Incorporation

Jiabei Zhou,[1,2] *Xianzhu Zhong,*[1] *Kenji Takada,*[1,3] *Maiko K. Okajima,*[2] *Masayuki Yamaguchi,*[1] *and Tatsuo Kaneko*[1,2]*

[1] Graduate School of Advanced Science and Technology, Japan Advanced Institute of Science and Technology (JAIST), 1-1 Asahidai, Nomi 923-1292, Japan

[2] Key Laboratory of Synthetic and Biological Colloids, School of Chemical and Material Engineering, Jiangnan University, 1800 Lihu Ave., Wuxi 214122, China

[3] Graduate School of Organic Materials Science, Yamagata University, 4-3-16, Jonan, Yonezawa, 992-8510, Japan

*To whom correspondence should be addressed; tkaneko@jiangnan.edu.cn

(correspondence affiliation is Jiangnan University)

Contents

Figure S1. FT-IR spectra of ABPBI films with various air compositions.

Figure S2. Air compositions calculation from the added silica nanospheres content.

Figure S3. Linear height analysis of the porous ABPBI film with 16% air composition.

Figure S4. SEM images, EDS mappings, and elemental analysis of pure ABPBI film.

Figure S5. SEM images, EDS mappings, and elemental analysis of porous ABPBI film with 8% air composition (silica content 10 wt%).

Figure S6. SEM images, EDS mappings, and elemental analysis of porous ABPBI film with 24% air composition (silica content 30 wt%).

Figure S7. SEM images, EDS mappings, and elemental analysis of porous ABPBI film with 40% air composition (silica content 50 wt%).

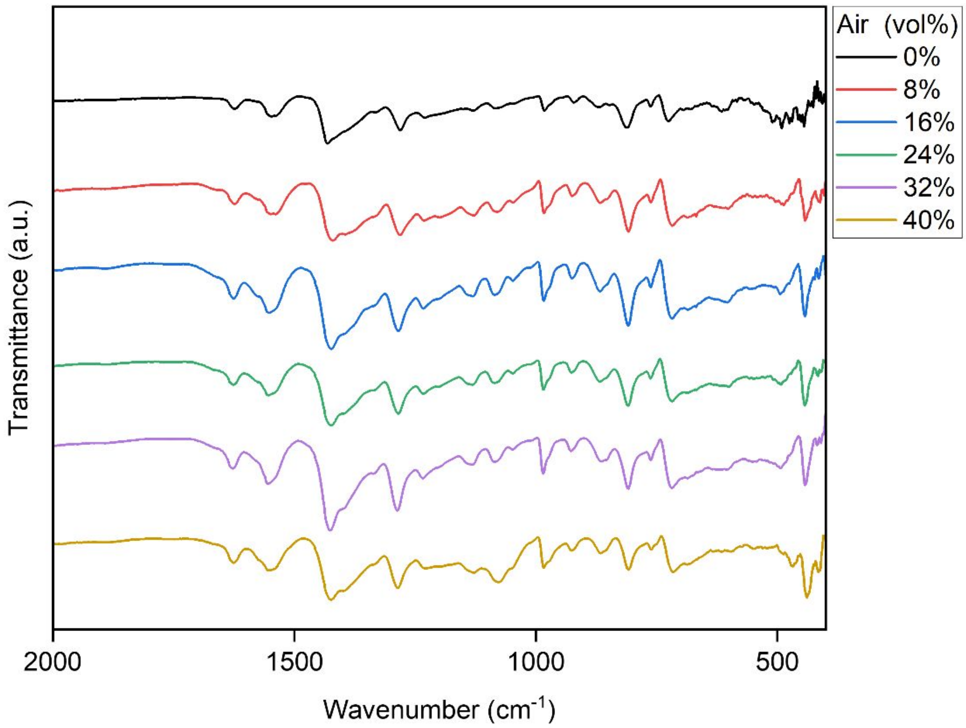

Figure S1. FT-IR spectra of ABPBI films with various air compositions.

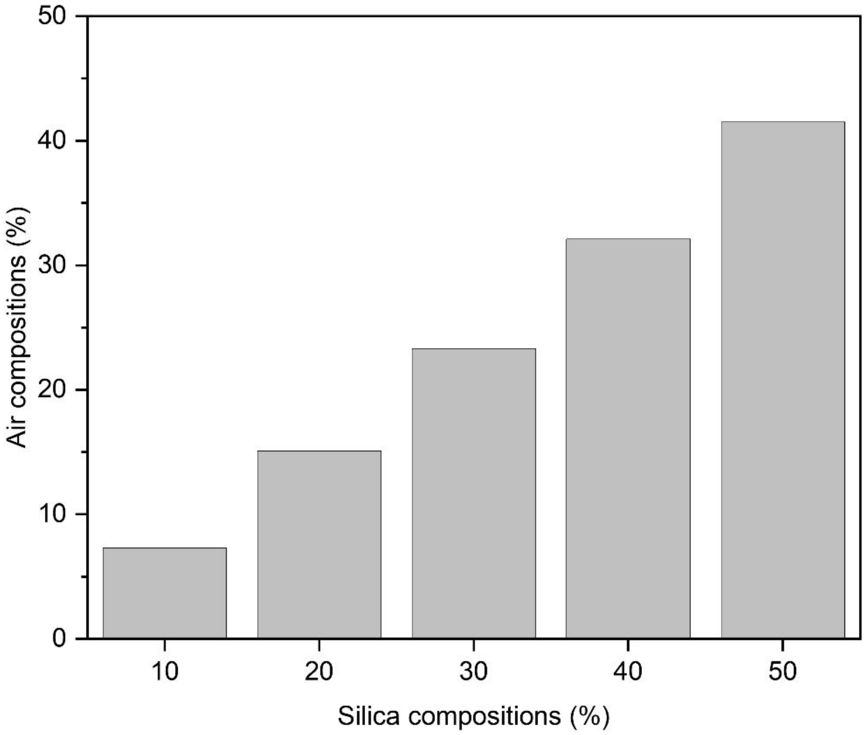

Figure S2. Air compositions calculation from the added silica nanospheres content.

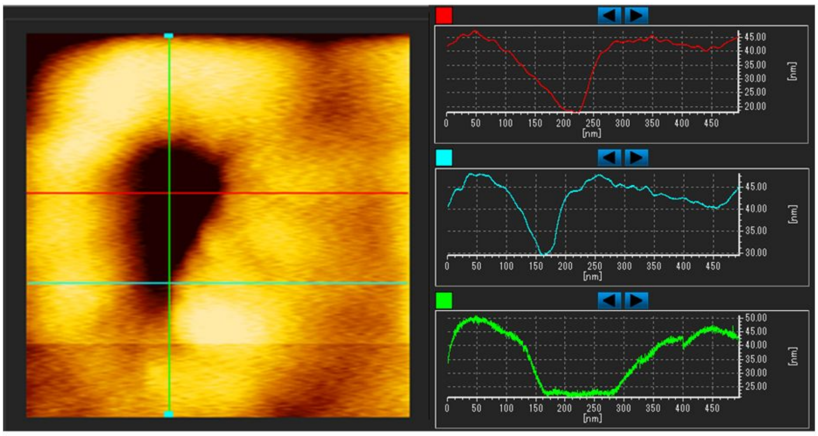

Figure S3. Linear height analysis of the porous ABPBI film with 16% air composition.

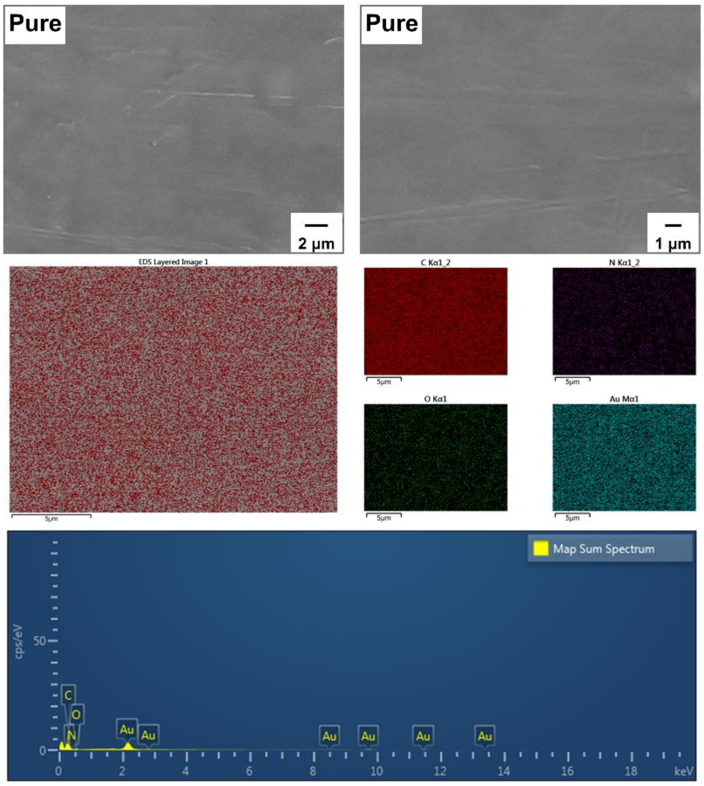

Figure S4. SEM images, EDS mappings, and elemental analysis of pure ABPBI film.

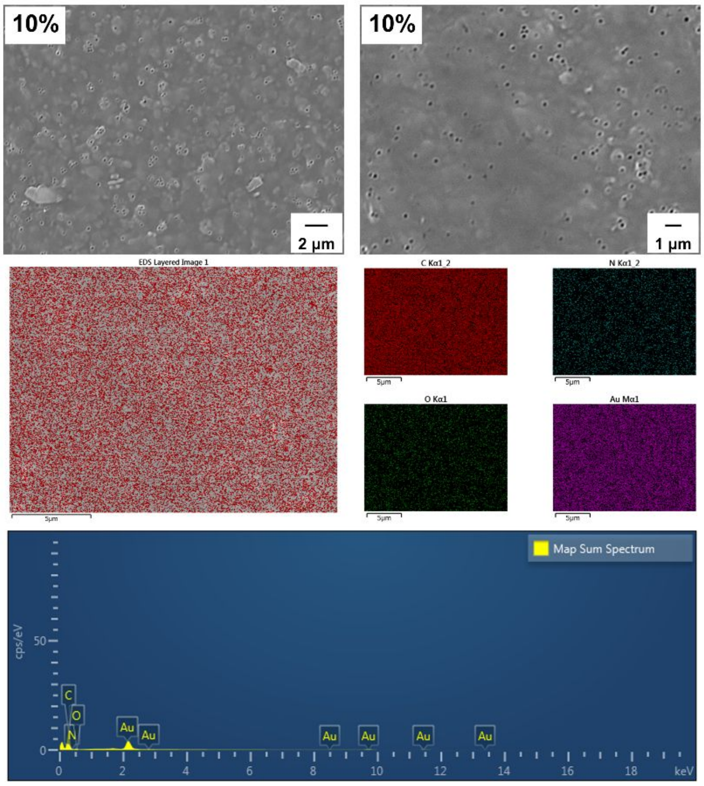

Figure S5. SEM images, EDS mappings, and elemental analysis of porous ABPBI film with 8% air composition (silica content 10 wt%).

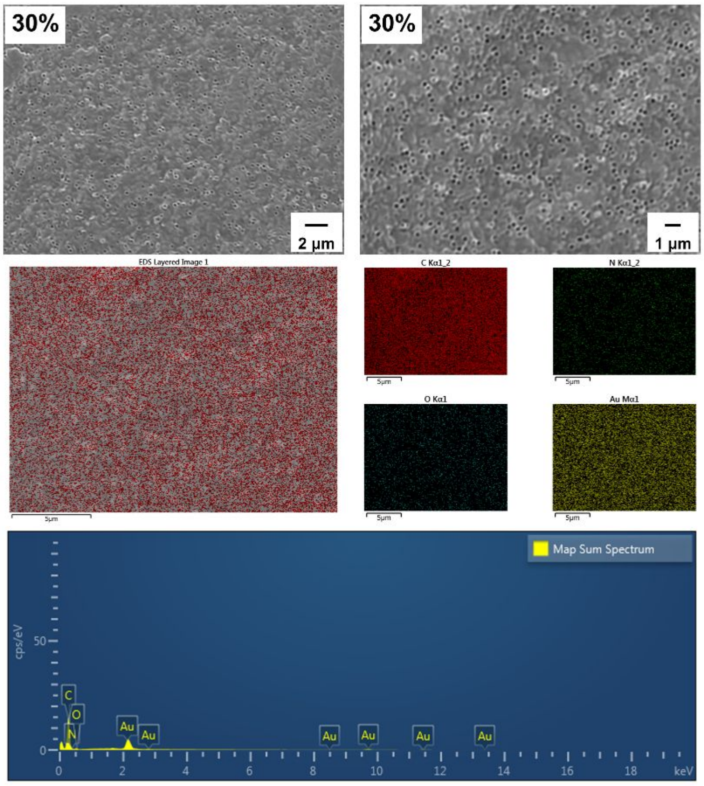

Figure S6. SEM images, EDS mappings, and elemental analysis of porous ABPBI film with 24% air composition (silica content 30 wt%).

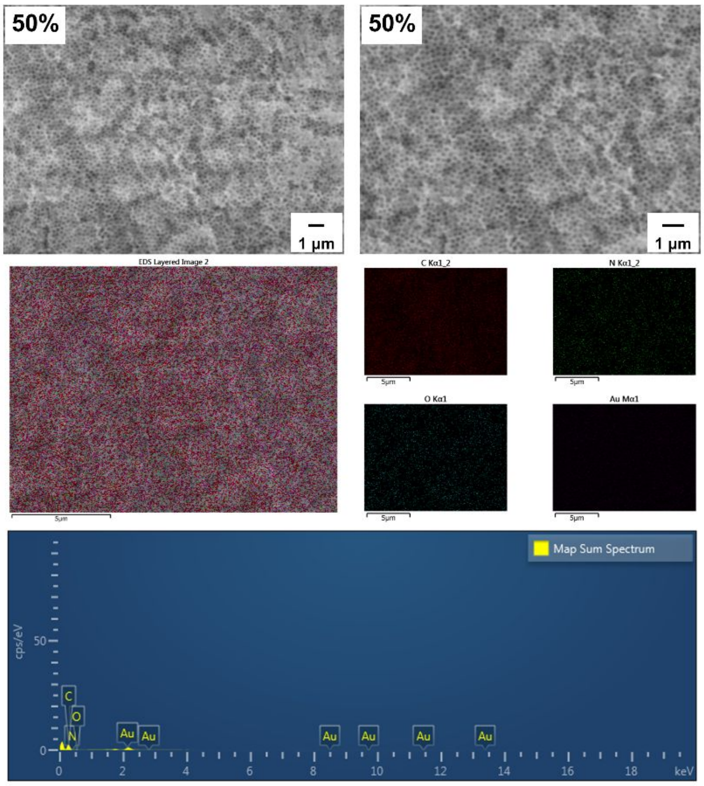

Figure S7. SEM images, EDS mappings, and elemental analysis of porous ABPBI film with 40% air composition (silica content 50 wt%).
